# Facial Painting and 3D Stereophotogrammetric Analysis of Facial Dynamics: A Reliable Anatomical Educational Method

**DOI:** 10.3390/jcm13082304

**Published:** 2024-04-16

**Authors:** Robin Pradel, Charles Savoldelli, Olina Rios, Philippe Kestemont, Barbara Lerhe

**Affiliations:** University Institute of Face and Neck, 31 Avenue de Valombrose, 06100 Nice, France

**Keywords:** body painting, dynamic anatomy, 3D skin vector, education, facial muscles, botulinum toxin

## Abstract

(1) **Background**: Accurate knowledge of the dynamic anatomy of facial muscles is crucial for the use of functional and aesthetic botulinum toxin injections. We studied the reliability and relevance of facial painting as a pedagogic tool for the dynamic anatomy of facial muscles. (2) **Methods**: Different facial expressions were performed by a female model after a professional makeup artist applied makeup to the various facial muscles on her left hemiface. A 3D photograph was taken at the beginning and end of each movement using the VECTRA H2 Imaging System device. Cutaneous movements were visualized using displacement vectors. The correlation between the theoretical and dynamic positions of the makeup–muscle was assessed by two facial anatomy experts, thanks to a correlation scale. (3) **Results**: The overall average score for the 11 analyzed muscles or muscle groups was 3.36 out of 4, indicating a “strong” to “very strong” estimated correlation. There was a moderate agreement between Evaluator 1 and Evaluator 2 (ICC: 0.64; 95%CI: [0.244; 0.852]; *p*-value: 0.005). (4) **Conclusions**: The educational model with facial makeup provides an indirect but nonetheless precise and reliable representation of all facial muscles on the skin’s surface. It is presented as a reliable and reproducible method, which exhibits great potential as a teaching tool.

## 1. Introduction

The use of botulinum toxin A (BoNT/A) for facial rejuvenation is considered the most widely practiced aesthetic medical procedure in the world [1]. It is safe and effective for reducing or even eliminating dynamic facial wrinkles [2]. Facial examination and injection planning are essential for achieving satisfactory results [3]. An in-depth clinical evaluation, including the diagnosis of expression lines [4], assessment of muscle contraction strength, and analysis of the anatomy of facial musculature, is crucial for developing an efficient, safe, and patient-specific injection protocol [1,5,6,7,8,9,10].

The initial and ongoing training of injectors in anatomy is essential and should be enriched with educational tools. Anatomy lessons with illustrations and cadaveric dissections are excellent methods for studying facial anatomy [11]. Unfortunately, these learning methods do not establish a direct link between muscles and dynamic facial expression wrinkles, as the subjects are, by nature, static [11,12].

Over the past decade, many medical faculties have introduced innovative methods alongside traditional anatomy studies, enabling the exploration of dynamic, realistic living anatomy. Currently, we have witnessed the diversification of teaching materials. This includes three-dimensional animations and images, and anatomical models of organs or body parts printed in three dimensions, thanks to the increasingly widespread use of three-dimensional printers in university hospitals, and the still relatively uncommon use of body painting techniques [13,14,15,16,17,18,19,20,21,22]. Actually, these techniques are currently underutilized in the study and teaching of facial anatomy. This is why we aim to investigate the use of body painting, coupled with three-dimensional stereophotogrammetric analysis, as an innovative and didactic method for analyzing facial dynamics. The primary objective of this study is to assess the reliability and relevance of facial painting as a pedagogic tool for the anatomy of facial muscles. The secondary objectives are as follows:

– Understand facial motricity by studying the dynamic specifics of each muscle;
– Identify and evaluate asymmetries between the right and left sides during muscle contraction.

## 2. Materials and Methods

A healthy, voluntary female model with Fitzpatrick skin type 2, aged 20, was selected in March 2023 at the University Institute of the Face and Neck at Nice (31 Avenue de Valombrose, 06100 Nice).

The exclusion criteria for the model were as follows: prior injection of BoNT/A or fillers, facial surgical intervention, central or peripheral facial paralysis, neurodegenerative diseases, and the presence of skin imperfections or facial tumors that would impede a rigorous analysis of wrinkles and facial movements.

The patient provided informed consent by signing a consent form after being informed about the procedure and the use of her images for research and educational purposes. A professional makeup artist, specializing in body painting techniques, was previously trained by the principal investigator of this study, an experienced maxillofacial surgeon. She was tasked with accurately replicating a schematic depiction of facial muscles on the left hemiface onto the skin.

The makeup artist and the model had not met before the session.

On our clean-skinned model, the professional makeup artist drew the facial muscles on the left hemiface in a single session, following the exact training she had received. The right hemiface served as a control, allowing for the concurrent analysis of skin wrinkles on the makeup-free skin.

All drawings were created on clean skin using professional makeup products, specifically water-based paints of the Paradise Makeup AQ brand by Mehron.

The various muscles that were drawn were the following: frontalis, temporalis, corrugator supercilii, procerus, orbicularis oculi, nasalis, levator labii superioris alaeque nasi, levator labii superioris, zygomaticus minor, zygomaticus major, depressor nasi septi, levator anguli oris, risorius, depressor anguli oris, depressor labii inferioris, mentalis, masseter, buccinator, and platysma.

**Figure 1:**
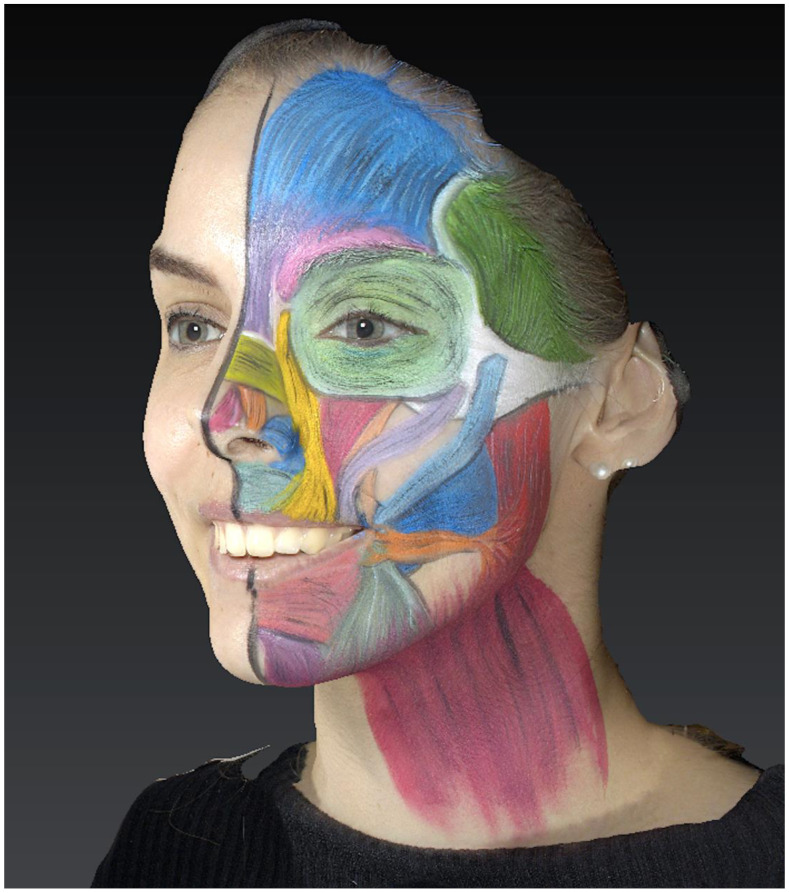
Three-dimensional photograph showing the painted muscles.

The muscles were depicted while adhering to their theoretical origin and insertion points, as well as the dimension and direction of their fibers (Figure 1).

A total of 30 photographs were captured using the Vectra H2 Imaging System camera device (Canfield Scientific, Inc., Fairfield, NJ, USA).

Initially, the model was photographed at rest, with a neutral expression on her face (static images).

Subsequently, the model was instructed to perform various facial expressions to induce facial wrinkles:

1. Smile: a broad, exaggerated smile with an open mouth showing teeth.
2. Anger/irritation: frowning and partial closure of the eyelid fissures.
3. Surprise: raised eyebrows, presence of forehead wrinkles, and widening of the eyelid fissures.
4. Rage: the model was asked to scream with furrowed brows and partial closure of the eyelid fissures.
5. Sadness: downturned corners of the mouth and eversion of the lower lip.

**Figure 2:**
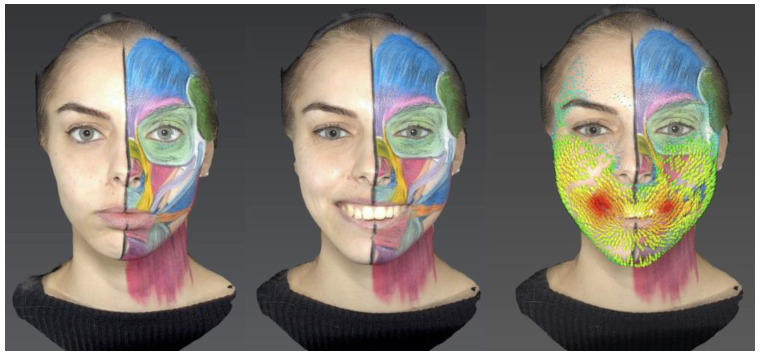
Neutral expression (**left**), smile expression (**center**), and reconstruction with skin displacement vectors (**right**).

Using Vectra Mirror Suite software, two-dimensional and three-dimensional facial expression videos were generated by using the static photos and the images taken at the end of each facial expression. Each expression image was superimposed onto its static image to analyze differences in skin position and displacement vectors (Figure 2).

Local changes in skin displacement were calculated using the automated algorithms of the Vectra Mirror Suite software and visualized using color and arrow size. Skin displacement progressively increases from blue to green, yellow, orange, and finally red, with the displacement proportional to the length of arrows of the same color. The main data collected from the left hemiface through 3D photographs included the following:

– Theoretical position of the muscle as applied by the makeup artist;
– Dynamic morphological anatomy of the muscle analyzed through skin movement vectors;
– Presence or absence of asymmetry compared to the non-makeup side.

The correlation between the theoretical and dynamic positions of the makeup–muscle was assessed by two facial anatomy experts, external to this study. We aimed for these evaluations to be conducted by experienced maxillofacial surgeons with expertise in medicine and facial anatomy. They evaluated the “real” muscle position based on the analysis of skin movements and skin displacement vectors. The evaluators rated the degree of correlation on a scale from 1 to 4, corresponding to no correlation, low correlation, strong correlation, or very strong correlation. The absence of skin movement and, therefore, movement vectors making correlation assessment impossible was scored as “UN” for “unscannable” (Table 1). 

Inter-observer agreement was assessed using the intra-class correlation coefficient (ICC).

## 3. Results

The main results and observations are presented here by muscle or muscle group (Table 2). Out of the 13 studied muscles or muscle groups, 2 were unscannable (UN) according to the evaluators, which represents 15.4% of the muscles studied.

The overall average score for the 11 analyzed muscles or muscle groups was 3.36 out of 4, indicating a “strong” to “very strong” estimated correlation. The average score given by Evaluator 1 was 3.18, and the average score given by Evaluator 2 was 3.54. There was a moderate agreement between Evaluator 1 and Evaluator 2 (ICC: 0.64; 95% CI: [0.244; 0.852]; *p*-value: 0.005).

## 4. Discussion

In total, 13 facial skin muscles or muscle groups of facial mimicry were studied. We made the decision not to apply makeup to the orbicularis oris muscle. It would have hindered our ability to study in precise detail the action and dynamic anatomy of the perioral and pericommissural muscles. This muscle can be studied specifically in a future study.

Two makeup muscles could not be analyzed, due to the absence of skin movement and, consequently, the absence of vectors resulting from their contraction (nasalis, depressor nasi septi).

The average correlation between facial makeup and dynamic anatomy, rated as strong to very strong, allows us to assert that facial makeup is a reliable and effective learning method for teaching the anatomy of facial skin muscles.

According to the results of our study, the makeup of facial skin muscles, under the supervision of a professional and specialist in facial anatomy, provides a very good approximation of real anatomy and can be reproducible despite anatomical differences among individuals.

A 2021 Korean study focused on 3D facial dynamics using a makeup model [23], and our results align with theirs. Furthermore, our work provides new insights into the use of facial makeup as a teaching method, as we evaluate the reliability and relevance of this technique, thanks to the correlation scale used by the evaluators, which had not been done before.

Furthermore, our study differs from this Korean study because we drew the muscles on only one side of the face. This allowed for a comparative analysis with the contralateral side, which, without makeup application, was exclusively subjected to standard visual examination. The non-makeup side thus served as a visual control, allowing for the evaluation of facial wrinkles during movement.

Over the past 20 years, new methods for learning anatomy have emerged (imaging, animations, and 3D reproduction), enabling a multimodal approach to the anatomical sciences.

Access to anatomy laboratories can sometimes be limited in hospitals and faculties due to the decrease in body donations in recent years, a reduction in resources, and the time allocated to this type of education. This limitation consequently hampers students and practitioners from conducting anatomical dissections.

In part, these issues have led to the emergence of new modalities for teaching anatomy [24].

Body painting has already been validated as a teaching method in many medical and surgical specialties. This method has proven effective in enhancing students’ memorization of presented anatomical structures. Body painting is not a new technique; it has been used by various cultures for centuries as a form of collective identity and during ritualistic activities [25].

This playful and didactic technique allows students and young practitioners to comprehend and, therefore, “treat” living, realistic, and dynamic anatomy [13,14,15,19,22]. 

For the observer, facial makeup provides the opportunity to visualize all the facial muscles involved in facial expression, working together in a dynamic manner, and their influence on the formation of facial wrinkles.

In the teaching of anatomy, three-dimensional printing and three-dimensional animation techniques have allowed for the analysis and understanding of anatomical movements in three-dimensional space. However, these techniques have limitations, particularly for the study of facial anatomy and skin movements and wrinkles, which can only be envisioned or approximated by these educational tools.

Compared to the use of three-dimensional animations and printed three-dimensional models in teaching facial anatomy, body painting offers several advantages. Indeed, the makeup can be adapted and modified in real-time if needed, during the teaching sessions. Furthermore, it allows the study of truly living anatomy, enabling the analysis of various facial muscles during facial mimic movements, which are known to activate several muscles simultaneously. Real-time analysis of wrinkles and skin movements allows for a much more relevant understanding of the action of each muscle and the role it plays in the formation of skin folds. This teaching technique also has the advantage of being extremely didactic, with significant interest reported by the students themselves [14,18,20,21]. 

Three-dimensional (3D) vector skin displacement analysis has been demonstrated to provide an objective quantification of skin movement [26,27].

This tool has a significant contribution, especially for complementing facial analysis and revealing subtle asymmetries that may not be easily visible during a standard clinical examination. It offers information on the strength, direction, and area of muscle contraction through the quantification of skin displacement.

These vectors indirectly reflect the underlying anatomy, though they do not allow for its precise and exact analysis.

Of course, to study a patient’s actual anatomy, one would need to use imaging techniques that enable precise and detailed analysis of soft tissues, such as MRI. The goal here was not to replicate the exact anatomy of the model but to create an indirect representation that is non-aberrant and as suitable as possible.

Facial makeup allows us to approximate both the resting anatomy and, more importantly, the dynamic anatomy of the muscles. This method is applicable to teaching the techniques of botulinum toxin A injection. Indeed, one can envision facial makeup sessions where the correct and expected positioning of the syringe and needle used for injections is represented for each targeted muscle of the treatment, based on dynamic anatomy and the specifics of the makeup model. This represents a significant advantage compared to digital animations, where we can visualize moving anatomy but cannot touch, move around the model, interact with the model or the students, etc.

Drawing the muscles on only one hemiface allowed for a comparative analysis with the contralateral side, which, without makeup application, is exclusively subject to standard visual examination. The non-makeup side serves as a control and enables the assessment of facial wrinkles during movement.

This aspect of our makeup application is especially valuable during teaching sessions, as it facilitates the explanation of the roles played by different muscles in the formation of facial wrinkles.

### 4.1. Asymmetries

**Figure 3:**
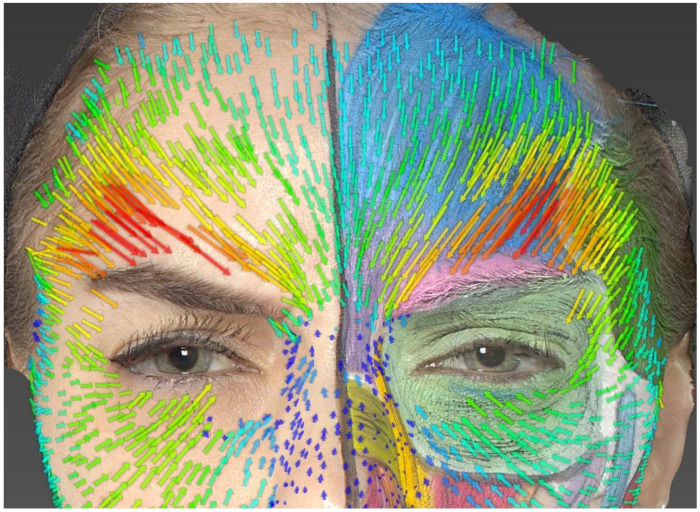
Anger expression. We can observe asymmetry in skin displacement between the right and left sides, particularly in the lateral part of the eyebrows.

Within the same muscle, the extent of skin displacement could differ depending on the part of the muscle involved, particularly in the corrugator muscle (Figure 3). Moreover, differences in the extent and size of skin displacement were observed between the same muscle groups on the left and right sides of our model, notably in the frontalis and corrugator muscles. These differences are crucial to consider during the facial evaluation of the patient and when formulating the BoNT/A injection plan [28,29].

### 4.2. Frontalis

We decide to present here, in an exhaustive way, the results of the most relevant muscles for a therapeutic perspective. Indeed, the frontalis, the corrugator supercilii, and the orbicularis oculi muscles all have FDA approval for botulinum toxin injections.

**Figure 4:**
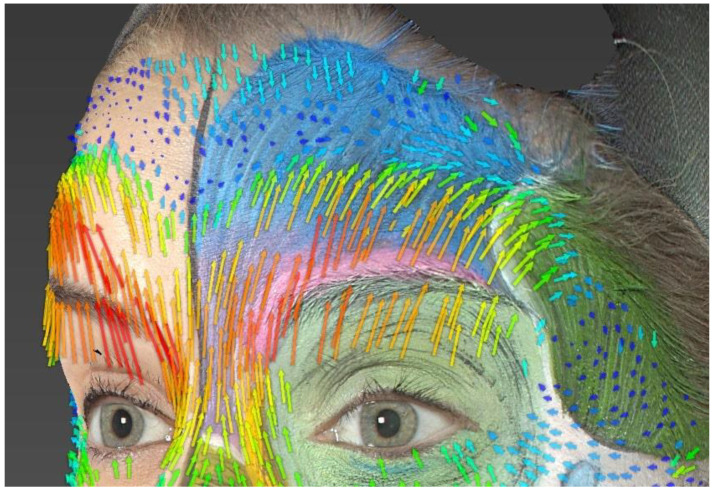
Analysis of the frontalis. Skin displacements vectors during “surprise expression”.

Another area of concern involves the lateral extent of the effect of the frontalis muscle. Skin displacement is observed laterally beyond the outer boundary of the drawn frontalis muscle. This can be explained by the forces exerted on the adjacent skin during muscle contraction, especially through skin tension lines. However, here, we observe a change in the direction of vectors and a clear lateral skin displacement compared to the theoretical outer limits of the drawn muscle. Thus, it is easily understood that in our model, the frontalis muscle likely extends more laterally than the drawn theoretical boundary (Figure 4).

It is noticeable that skin displacement primarily occurs in the lower part of the forehead. Furthermore, bi-directional skin movements are observed with a transition line approximately located at the midpoint of the forehead. This corresponds to one of the three models of skin displacement on the forehead [27].

**Figure 5:**
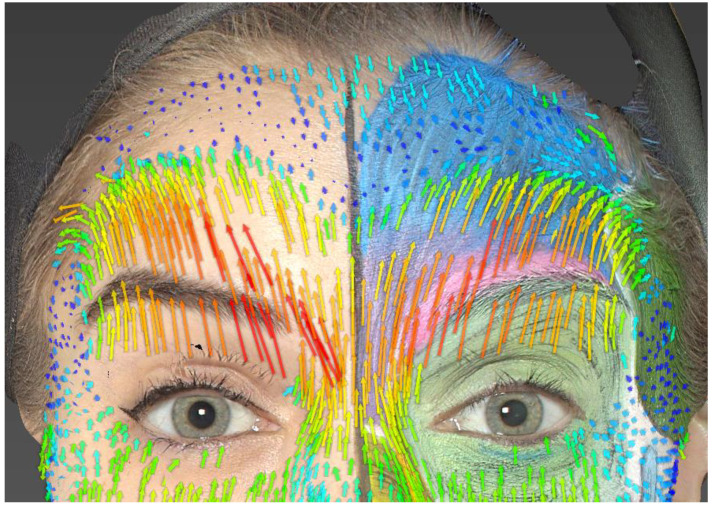
Analysis of the frontalis. Asymmetry in the elevation of the eyebrows.

The right eyebrow is raised higher than the left at the end of the movement, especially in its lateral part, which is also evident in the study of movement vectors (predominantly red and orange vectors, longer than those on the left). We can deduce that there is a difference in contraction force between the left and right frontalis muscles of our model (Figure 5).

Therefore, when treating forehead wrinkles, it is important to study the action of the frontalis muscle and the resulting frontal skin displacement to propose an injection scheme tailored to the patient. The goal is to avoid the occurrence of undesirable and aesthetically disappointing results, such as the “Spock eyebrow” (excessively raised eyebrow tail) or the “Mephisto effect” [30].

### 4.3. Corrugator Supercilii

Regarding the corrugator supercilii, the orientation of the drawn muscle fibers partially matches the orientation of the vectors. Indeed, the drawn muscle is thin and horizontal above the eyebrow, while the vectors are oriented medially and inferiorly. It is understood that the corrugator muscle in our model is likely wider and has higher insertions located above the eyebrow, which can be inferred from the study of the wrinkles formed on the patient’s right hemiface. The area of the most significant skin displacement is located at the junction of the medial third and lateral third above the eyebrow. Furthermore, an asymmetry in skin displacement is visible through the study of the vectors (greater skin movements on the right than on the left).

### 4.4. Orbicularis Oculi

Concerning the orbicularis oculi muscle, it is noted that the skin displacement vectors are present very laterally compared to the outer boundary of the drawn muscle. It is understood that the actual anatomical muscle is likely more extensive laterally than what is represented with makeup.

## 5. Conclusions

The educational model with facial makeup provides an indirect but nonetheless precise and reliable representation of a large majority of facial muscles on the skin’s surface. It is presented in this study as a reliable and reproducible method, which, in a demonstrative and highly didactic manner, exhibits great potential as a teaching tool, particularly for botulinum toxin type A injection techniques.

## Figures and Tables

**Table 1:** Correlation scale.

Correlation scale	UN	1/4	2/4	3/4	4/4
	Unscannable	Zero correlation	Low correlation	Strong correlation	Very strong correlation
	Absence of skin displacement vectors	<20% of vectors aligned with the makeup-applied muscle fibers	20–50% of vectors aligned with the makeup-applied muscle fibers	50–80% of vectors aligned with the makeup-applied muscle fibers	>80% of vectors aligned with the makeup-applied muscle fibers

**Table 2:** Results per muscle.

	Evaluator 1	Evaluator 2	Average Score per Muscle (out of 4)
Frontalis	3	3	3
Procerus	3	4	3.5
Corrugator supercilii	4	4	4
Orbicularis oculi	2	2	2
Nasalis	UN	UN	UN
LLSAN	4	4	4
Levator labii superioris	4	4	4
Zygomaticus major	4	4	4
Levator anguli oris and Zygomaticus minor	3	4	3.5
Risorius	3	3	3
Depressor anguli oris and Depressor labii inferioris	4	4	4
Mentalis	1	3	2
Depressor nasi septi	UN	UN	UN
Average score per evaluator (out of 4)	3.18	3.54	3.36

## Data Availability

The original contributions presented in the study are included in the article, further inquiries can be directed to the corresponding author.
